# In Silico Screening of Available Drugs Targeting Non-Small Cell Lung Cancer Targets: A Drug Repurposing Approach

**DOI:** 10.3390/pharmaceutics14010059

**Published:** 2021-12-28

**Authors:** Muthu Kumar Thirunavukkarasu, Utid Suriya, Thanyada Rungrotmongkol, Ramanathan Karuppasamy

**Affiliations:** 1 Department of Biotechnology, School of Bio Sciences and Technology, Vellore Institute of Technology, Vellore 632014, India; muthukumar.t@vit.ac.in; 2 Program in Biotechnology, Faculty of Science, Chulalongkorn University, Bangkok 10330, Thailand; noteutidii@gmail.com; 3 Biocatalyst and Environmental Biotechnology Research Unit, Department of Biochemistry, Faculty of Science, Chulalongkorn University, Bangkok 10330, Thailand; 4 Program in Bioinformatics and Computational Biology, Graduate School, Chulalongkorn University, Bangkok 10330, Thailand

**Keywords:** drug-repositioning, MEK inhibitor, MM/GBSA, Glide docking, MD simulation, MM/PBSA

## Abstract

The RAS–RAF–MEK–ERK pathway plays a key role in malevolent cell progression in many tumors. The high structural complexity in the upstream kinases limits the treatment progress. Thus, MEK inhibition is a promising strategy since it is easy to inhibit and is a gatekeeper for the many malignant effects of its downstream effector. Even though MEK inhibitors are under investigation in many cancers, drug resistance continues to be the principal limiting factor to achieving cures in patients with cancer. Hence, we accomplished a high-throughput virtual screening to overcome this bottleneck by the discovery of dual-targeting therapy in cancer treatment. Here, a total of 11,808 DrugBank molecules were assessed through high-throughput virtual screening for their activity against MEK. Further, the Glide docking, MLSF and prime-MM/GBSA methods were implemented to extract the potential lead compounds from the database. Two compounds, DB012661 and DB07642, were outperformed in all the screening analyses. Further, the study results reveal that the lead compounds also have a significant binding capability with the co-target PIM1. Finally, the SIE-based free energy calculation reveals that the binding of compounds was majorly affected by the van der Waals interactions with MEK receptor. Overall, the in silico binding efficacy of these lead compounds against both MEK and PIM1 could be of significant therapeutic interest to overcome drug resistance in the near future.

## 1. Introduction

Lung cancer accounts for about a quarter of all cancer deaths, among them 82% of deaths were being caused by intentionally smoking cigarettes. The development of advanced therapies for the management of the early and metastatic stages of lung cancer were not yet discovered over the past 40 years. Although some treatment measures are available to control the earlier stages of lung cancer, poor outcomes reduce the overall patient survival rates. One of the common clinical symptoms of lung cancer is frequently coughing for a particular period. For example, patients in the United States who had been coughing for three weeks were finally identified with lung cancer [1]. In the United Kingdom, smoking is responsible for 71% of lung cancer deaths, whereas 1% of the deaths of passive smokers were reported. The Canadian researchers reports that the lung cancer deaths in smokers were 15% higher than in non-smokers. In India, 9.3% of the cancer deaths were associated with lung cancer, containing both male and female patients [2]. The low lung cancer survival rates reflect the high number of patients diagnosed with metastatic disease (57%). Currently, surgery, radiation therapy, chemotherapy and targeted therapies were used to treat the lung cancer patients. Among these methods, targeted therapies demonstrated better outcome during the cancer treatment [3,4]. Genetic expression and mutational studies were certainly used to identify the definitive target for lung cancer. The incidence of particular mutations varies depending on ethnicity and location. The EGFR mutations that were reported in Caucasians were found to be 10%, whereas 60% of the mutational rates were reported in Asian people [5]. Ultimately, the tyrosine kinase pathway plays a major role in the tremendous increase in lung cancer deaths. Mitogen-activated protein kinase (MAPK) is one of the promising growth signaling pathways. The aberrant activation of this pathway’s intermediates leads to uncontrolled cell growth and differentiation. In many cancer types, concomitant mutations occurred in RAS and BRAF, which is the reason for the consecutive activation of ERK, which is responsible for the activation of many transcription factors [6]. Hence, targeting the pathway receptors using checkpoint inhibitors leads to effective therapy in most cancers. However, a strong association between RAS and GTP impedes the direct inhibition of RAS. The lack of understanding regarding the allosteric sites is also a hindrance to the development of RAS targeting inhibitors [7]. The next intermediate RAF is another important target when there is an existence of BRAFV^600^ mutations. Nevertheless, the acquired resistance in RAF selective inhibitors is the reason for the constant activation of the MAPK pathway in many cancers [8]. Therefore, it is possible to affirm a downstream cut-off of the MAPK pathway at a protein kinase called MEK. The MEK receptor is a key node in the MAPK pathway, which is the only known substrate of its downstream effector, ERK. In the recent decade, hundreds of MEK inhibitors were discovered to target the allosteric binding site of MEK [9]. Although these selective inhibitors were effective at the allosteric site, a poor cytotoxicity profile limits their treatment progress. For instance, the most potent kinase inhibitors, such as binimetinib, selumetinib, cobimetinib and rafametinib, caused diarrhea, elevated lipase levels and rashes as adverse effects [10,11]. It is important to note that a recently approved MEK selective inhibitor trametinib showed the most efficacy in the BRAF mutant tumors in combination with dabrafenib [12,13]. However, trametinib alone showed additional side effects during the treatment period in non-small cell lung cancer patients. For instance, trametinib specifically affects the ocular region of the patients. Moreover, a severe complication in the ocular region may lead to permanent vision loss in the patients [14]. In addition to their toxic effects, several MEK inhibitors were resistant to the BRAF mutations through the activation of adjutant signaling pathway receptors. The initial solution to the problem of resistance to therapy is the dual inhibition of crucial targets with the administration of a single therapy. It is also interesting to note that the inhibition of multiple kinases will produce better outcomes during clinical trials. For instance, the combination of MEK and JAK2/STAT3 pathway inhibition reduces the potential impact on drug resistance in colon cancer [15]. Similarly, a combination of MEK and PI3K inhibitors is a powerful treatment option for NSCLC patients who have developed resistance to EGFR–TKIs [16]. Note that dual inhibitors will produce more beneficial effects than the combined inhibitors in terms of cost and time taken for the approval. Note also that PIM1 is a critical effector facilitating cross-talk across several neighboring pathways, in particular to the MAPK pathway. Recent studies highlight that MEK inhibitors lead to the increased expression of PIM1, thereby increasing cancer cell growth [17,18]. Keeping this in mind, we framed an in silico-based drug repurposing workflow to screen the potential inhibitors that act against both MEK and PIM1.

Drug repurposing has become one of the most popular ways for increasing the efficiency and cost-effectiveness of drug development. Importantly, the discoveries of the novel indications of existing drugs were the major applications in drug repurposing strategies. In recent years, almost 30% of the FDA-approved drugs and vaccines were discovered by in silico approaches. For instance, the discovery of zanamivir was made possible using a computer-aided drug design technique based on the crystal structure of influenza virus neuraminidase [19]. Adding together the implementation of machine learning principles and virtual screening would certainly enhance the accuracy of screening results. Machine-learning-based approaches produce more reliable results and provide faster outcomes by learning existing experimental data [20]. Hence, we incorporated machine-learning-based scoring functions (MLSF) to screen the potential compounds against the MEK receptor since they have attained a plateau in their performance during the binding affinity prediction [21]. We are certain that the outcome of this study is of immense importance for the experimental biologist involved in the screening of MEK inhibitors.

## 2. Methodology

### 2.1. Dataset

Structural information of proteins and ligand molecules were retrieved from the protein data bank (PDB) and DrugBank database, respectively. The 3D structure of two protein molecules, such as MEK1 (PDB ID:3W8Q) and PIM1 (PDB ID: 5KZI), were downloaded in the PDB format [22,23]. Eventually, the DrugBank molecules were downloaded as three subsets containing FDA (Food and Drug Administration)-approved drugs (*n* = 3085), experimental drugs (*n* = 5689) and investigational drugs (*n* = 3034) for screening application.

### 2.2. Protein and Ligand Preparation

Preparation of the receptor molecules was carried out using protein preparation wizard present in the maestro workspace. The four major pre-processing steps that were carried out include: (i) bond order assignment, (ii) addition of missing hydrogen atoms, (iii) creation of zero-order bond for the metal atoms and (iv) di-disulfide bond creation. The pre-processed proteins were then subjected to a hydrogen bond optimization process. During the optimization, the protonation state of each amino acid residue was calculated, and the pH was adjusted to 7 ± 0.5 using the predicted pKa values. The predicted and adjusted pKa values of amino acid residues of the proteins were presented in Supplementary Materials Table S1. If the predicted pKa was less than pH value, the amino acid functional groups were protonated during the optimization process. On the other hand, if the pKa was greater than pH value, the deprotonation process took place in those amino acid functional groups. Note that, if the pKa and pH values were equal, 50% protonation and 50% deprotonation took place. Subsequently, the excess water molecules were removed because of the higher occupancy at the receptor binding pocket. Finally, the heavy atoms were converged at the RMSD (root-mean-square deviation) value of 0.30 Å using restrained minimization process [24].

The ligand molecules were processed using LigPrep module in the maestro workspace. Initially, all the ligand molecules were subjected to energy minimization using OPLS_2005 (optimized potentials for liquid simulations) force field at pH 7.0 ± 2. To avoid stereoisomer formation, the chiral centers of all the ligand molecules were chosen to preserve their original state. Notably, all the ligand molecules were allowed to generate only one structural conformation.

### 2.3. Binding Site Analysis and Grid Generation

Binding site prediction and pocket druggability analysis are the few important perquisites in drug repurposing strategy [25,26]. Here, we used the sitemap algorithm to predict the binding as well as druggable pockets present is the target receptor. Sitemap predicts the hot spots based on the number of hydrogen bond donors and acceptors, hydrophobic atoms and the concave sites present in the receptor [27]. Later, the grid generation was executed by using receptor grid generation wizard. The grid box was generated around the predicted hot spot residues with the partial charge cut-off of 0.25 and a scaling factor of 1.

### 2.4. Glide Docking and MM/GBSA Analysis

All prepared ligand molecules were screened through the high-throughput virtual screening (HTVS) method followed by being docked into the predicted binding sites using Glide XP (Extra-precision) protocols. We have utilized a flexible docking method with the van der Waals radii scaling factor of 1 Å to soften the receptor binding site. The atoms of the protein with partial charges less than or equal to 0.25 were scaled with a van der Waals scale factor of 0.8 [28]. Later, the ligand interaction diagram was visualized for the in-depth understanding of ligand contacts with the target receptor. Further, the docking score was revalidated by the binding free energy calculations using Prime-MM/GBSA (molecular mechanics with generalized born surface area) analysis. XP docked complexes were further subjected to minimization at the local optimization feature with the force field of OPLS_2005. Prime estimates the binding free energy by comparing the energy of the complex state to the energy of the individual protein and ligand molecules [29].

### 2.5. Scoring Functions

#### 2.5.1. RF-Score Analysis

The MLSF analyzes the molecular docking outputs of the existing protein–ligand complex to predict the binding affinity of unknown compounds [30]. Here, we used RF-Score-VS, which uses a random forest algorithm to predict the binding affinity of the molecules. It is a standalone program (https://github.com/oddt/rfscorevs, accessed on 3 June 2021) that was implemented using the ubuntu terminal. In this tool, a random forest model was robustly set to generate a maximum number of 500 trees. It is worth noting that random forest model used in this study implicitly captures binding effects that are hard to model explicitly. Protein and ligand molecules were supplied in sdf and pdb format, respectively, for the RF score calculation.

#### 2.5.2. Tanimoto Coefficient Calculation

Tanimoto coefficient is one of the most important similarity measures during the virtual screening process [31]. BulkTanimotoSimilarity() function in the RDKit package gets a fingerprint query and a collection of fingerprints to display the list of similarity results for each fingerprint target. This metric estimates the proportion of the common bits in the range of 0 to 1 between the chemical fingerprints. In this section, the Tanimoto resemblances of all DrugBank compounds were tested against the fingerprints generated by the trametinib.

### 2.6. Molecular Dynamics (MD) Simulations

The complex structures of two focused compounds and the known drug from molecular docking were dynamically simulated by the near-physiological-motion MD simulations. The AMBER ff14SB force field and generalized AMBER force field version 2 (GAFF2) were employed to treat bonded and non-bonded interaction parameters of all simulated complexes [32]. The TIP3P water model [33] was used to solvate the system with minimum padding of 10.0 Å between the protein surface and the solvation box edge. Then, either sodium or chloride ions were randomly added to neutralize the overall charge of the molecular system. Minimization of the hydrogen atoms and water molecules was performed by using 500 steps of steepest descent (SD) followed by 1500 steps of conjugated gradient (CG) methods. All studied systems were proceeded to run under the periodic boundary condition with the isothermal–isobaric (NPT) scheme according to the previous studies [34,35,36,37,38]. The electrostatic interactions were treated by the particle mesh Ewald summation method [39], whereas The SHAKE algorithm [40] was used to constrain all covalently connected hydrogen atoms. The temperature was controlled by the Langevin thermostat [41] with a collision frequency of 2 ps^−1^ and gradually increased from 10 to 310 K. In addition, Berendsen barostat [42] was employed to control pressure with a relaxation time of 1 ps. Each simulated system was subsequently simulated under the NPT ensemble (310 K, 1 atm) until reaching 100 ns. The MD production for all systems was set to 100 ns by the 2-fs increment of a time step. The root-mean-square displacement (RMSD) and hydrogen bond (H-bond) occupations were calculated through the cpptraj module, while per-residue decomposition energy (${\Delta G}_{\mathrm{binding}}^{\mathrm{residue}}$) was estimated by MM/PBSA.py implemented in AMBER16.

### 2.7. End-Point Binding Free Energy Calculations

To evaluate the ligand-binding capability, the total binding free energy (Δ*G*_binding_) of each complex was estimated based upon the solvated interaction energy (SIE) approach [43]. In theory, Δ*G*_bind_ can be estimated as the summation of the van der Waals (*E*_vdW_), electrostatic (*E*_ele_(D_in_)), reaction field (Δ*G*_RF_(*ρ*,*D*_in_)), cavity (*γ*Δ*SA*(*ρ*)), and a constant (*C*) value, which was expressed as the following equation

Δ*G*_bind_ (*ρ*,*D*_in_, *α*, *γ*, *C*) = *α*[*E*_vdW_ + *E*_ele_(*D*_in_) + Δ*G*_RF_(*ρ*,*D*_in_) + *γ*Δ*SA*(*ρ*)] + *constant*

where *D*_in_ is the solute interior dielectric constant. *E*_vdW_ and *E*_ele_ are denoted as intermolecular van der Waals and Coulombic interaction energies in the bound state, respectively. Δ*G*_RF_ is the electrostatic polarization component of the solvation free energy to binding, and Δ*G*_cavity_ (*γ*Δ*SA*) represents the nonpolar contribution of the solvation free energy to the binding. The coefficients set to every calculation are α = 0.105, γ = 0.013 and C = −2.89.

## 3. Result and Discussion

### 3.1. Binding Site Prediction

**Figure 1:**
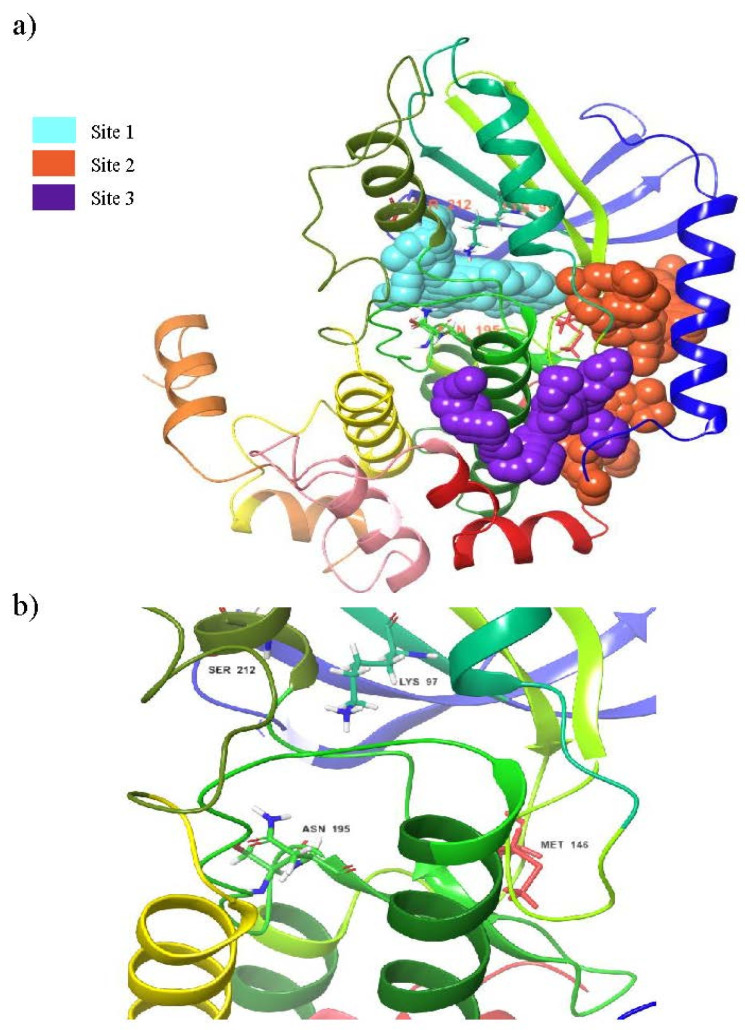
(**a**) Schematic representation of top three predicted binding sites. (**b**) Functionally important residues in site 1.

The identification and characterization of the druggable binding pocket of the MEK1 receptor were identified by employing the sitemap module. The best five binding sites of MEK1 and their physiological characteristics predicted by the sitemap were tabulated in Table 1. The larger quantity of hydrophobic residues at the top three sites shows improved pocket adaptation for the ligand binding. Notably, the druggability score of each pocket was in the range of 0.6 to 1. Sites 4 and 5 have a Dscore less than 0.7, which implies the poor druggability of those pockets. Whereas, sites 1, 2 and 3 have resulted in a Dscore of ~1, which indicates that these sites highly encourage the binding of drug-like molecules on their pocket residues [44]. Although the enclosure of site 3 (0.673) is lower, the higher Dscore (1.005) and sitescore (0.974) make the pocket suitable for molecule binding. The top three sites that displayed significant physiological characteristics for the binding of drug-like molecules are shown in Figure 1. Among these three binding sites, site 1 encompasses the end of the activation loop region where the substrate ERK binds to MEK. In addition, site 1 comprises the important amino acid residues for the activation of the MEK receptor and DGF motif, which is an important motif involved in the MEK phosphorylation process. In addition, site 1 comprises amino acid residues, such as VAL 127, SER212, LYS97, VAL211 and ATP binding site [45]. Since site 1 comprises the crucial pockets, we have utilized the results obtained from site 1 during the validation step and other analyses.

### 3.2. Validation of Molecular Docking

The validation of Glide XP docking and RF-Score-VS were accomplished by using external datasets (Table S2). The dataset consists of 25 active compounds and 75 decoy compounds against mitogen-activated protein kinase, which were randomly sampled from the Database of Useful Decoys-Enhanced (DUD-E) using the ‘sample()’ function in pandas to validate the docking and RF-Score-VS analysis. The results were incorporated into the maestro workspace for enrichment analysis [46]. The ‘enrichment calculator’ tool was used here to evaluate the screening process. On both of the screening analyses, the compounds were sorted by the respective scoring functions, for instance, the Glide XP score and RF-Score-VS_v2 for molecular docking and RF-Score-VS analysis, respectively. Later, the effectiveness of the screening methodologies to differentiate between the actives in the decoy set of compounds was tested by producing a receiver operating curve (ROC) (Figure S1). A total of 11 decoys were outranked during the screening process using RF-Score-VS. On the other hand, seven decoys were outranked during the molecular docking analysis. The smaller number of outranked compounds indicates the effectiveness of these screening algorithms. Further, these measures were evaluated using receiver operating characteristic curve (ROC) analysis. Importantly, the ROC value of docking and RF-Score-VS were 0.902 and 0.850, respectively. Moreover, the area under the curve (AUC) was calculated as 0.801 and 0.762 for molecular docking and RF-Score-VS, respectively. Since the AUC value of docking and RF-Score-VS are above 0.7, we believe that both algorithms have the potential to discriminate the active compounds from the target database. Further, we have accessed Pearson’s and Spearman’s correlations between the docking score and experimentally determined binding affinity of the 25 active compounds. It is worth noting that Pearson’s and Spearman’s correlation values of 0.758 and 0.818, respectively, were observed. All of these findings indicate that the lead compounds produced through these screening approaches may potentially be effective towards further experimental works.

### 3.3. Virtual Screening

A total of 11,808 molecules from the three subsets of Drugbank were screened through the HTVS docking method. Later, the screened hit molecules (n = 7075) were docked into the best predicted binding site, such as site 1, using the Glide XP method. Note that trametinib was used as a reference compound in all the analyses. The XP docking score of reference compound −3.423 kcal/mol in site 1 was then used as a threshold for further screening of hit molecules. Subsequently, the top 50% of the molecules resulting from the XP docking on site 1 were redocked to site 2 and site 3. A total of 3125 and 2813 compounds were predicted to bind better than the reference compound on site 2 and site 3, respectively. The results from the docking study were then integrated to eliminate the false positive compounds. The results indicate that 2468 compounds were able to bind tightly with all three binding sites predicted by the algorithm.

Recently, machine-learning-based scoring functions evolved to measure the binding affinity of the compounds with their multiple characteristic features. In particular, RF-Score-VS obtains a remarkable hit rate up to 88.6% throughout the DUD-E targets [21]. Hence, we analyzed the binding ability of all the screened hit compounds using RF-Score-VS. It is notable that the reference compound trametinib showed an RF-score of 6.565. Fortunately, a total of 5152 compounds were ranked better than the reference compound in RF-Score-VS analysis. The comparison of the docking study and RF-Score calculation yielded a total of 1654 compounds. These compounds were screened through the Tanimoto coefficient calculation using the rdkit package. All the compounds’ fingerprints were generated and tested for structural similarity against the reference compound. The calculations of the Tanimoto coefficients of the screened hit compounds were tabulated in Table S3. Here, we chose a Tanimoto coefficient of 0.6 as a threshold value for screening the compounds [47]. Overall, 368 compounds gained a Tanimoto coefficient value above 0.6, which will be taken for further screening studies.

### 3.4. MM/GBSA Analysis

Recent literature studies highlight that the total binding free energy values predicted during the MM/GBSA calculation correlate well with the experimentally measured biological activity [48]. Thus, Prime-MM/GBSA was implemented as a post-scoring process for the validation of the screened hit molecules. The pose viewer file generated during the Glide XP docking on site 1 was considered as an input file for this analysis. The results of the MM/PBSA studies on the top 15 hit compounds and their associated energy values were represented in Table 2. Moreover, the replicability of the binding affinity by Glide docking was evaluated through three-fold validation of XP docking on 15 hit compounds. The binding free energy values obtained during the three iterations were represented in Table S4. It is evident from the table that the 14 hit compounds were able to display a better docking score than the reference compound in all three docking processes. Although the docking score slightly differs during each docking simulation, the compounds ranking was most likely the same as the initial docking simulation. These results demonstrate the excellent consistency of the compounds ranking during the docking simulation. It is evident from Table 2 that the binding free energy values of the compounds varied from −46 to −87 kcal/mol. The available literature information depicts that lipophilicity and van der Waals energy were key factors for the proper binding of the ligand molecules with the target receptor [49,50]. It is evident from the table that the lipophilicity of the compounds DB12661, DB07642, DB01771 and DB07177 were highly favorable for the ligand binding. Although two compounds, DB01711 and DB07177, showed better lipophilicity, the minimal van der Waals interaction limits the total binding free energy of these compounds. 

It should be noted that, except DB02849 and DB04841, most compounds in terms of binding have been highly favored by van der Waals interaction energy. In particular, the compounds DB12661 and DB07642 displayed a massive van der Waals interaction energy value of −57.476 and −55.062 kcal/mol, respectively. Although these compounds show limited coulombic potential, the maximum contribution of van der Waals interaction energy is responsible for the tight binding of these compounds with the MEK1 receptor. Moreover, the total binding free energy values of these compounds, DB012661 and DB07642, were much higher (>−80 kcal/mol), which is also higher than the other compounds investigated in this analysis. Hence, we believe that the compounds DB012661 and DB07642 may more tightly bind with the MEK1 receptor than the other compounds screened in our analysis.

### 3.5. Structural Properties of Hit Compounds

The similarity between the ligand molecules was evaluated by mapping the pharmacophoric structure of the hit compounds. Here, we have used “2D structure alignment” utility present in the maestro workspace to align the structure of the compound. Moreover, we have predicted the ADME/T properties of the hit compounds using the QikProp module available in the Schrödinger package. These results were incorporated in Table 3. Note that these structures were aligned against the reference compound trametinib. Interestingly, four hit compounds, such as trametinib, DB08251, DB02849, DB04241 and DB12847, had pyridine as a common scaffold in their structures. Pyridine is an essential pharmacophore and an extraordinary heterocyclic system in the realm of anti-cancer drug development [51]. It is also noted that the hit compounds displayed acceptable ADME/T values during the QikProp analysis. The central nervous system activity prediction is one of the main properties during the ADME/T prediction [52]. All the compounds except DB12661 and DB07642 were exhibited at the in-active state, which is indicated by a CNS value of −2. Moreover, the other properties, such as stars (acceptable range: 0–5) and HOA (acceptable range: 1–3), were in the acceptable range in all the hit compounds.

### 3.6. Binding Mode Analysis

**Figure 2:**
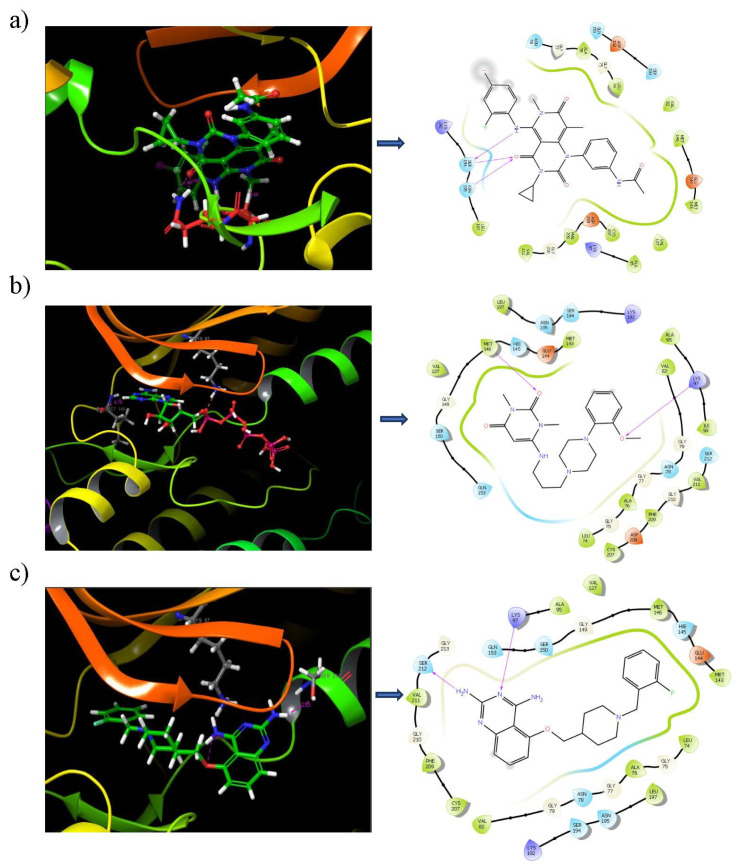
Ligand interaction diagram of the hit compounds. (**a**) Reference; (**b**) DB12661; (**c**) DB07642 with MEK1 receptor.

The binding frequencies of the top 14 compounds on the three different binding sites were represented in Figure S2. It is notable that the binding positions of the compounds at each binding site were more or less the same in site 1 and site 3. Since the binding site residues were dispersed larger in site 2, a few compounds, such as DB12661, DB02709, DB12847 and DB08251, were positioned differently from the other compounds. Most of the ligand molecules were bound tightly in site 1, as indicated by the better docking score in Table S3. Hence, the ligand binding conformations of the top hit compounds in site 1 were analyzed (Figure 2). It is evident from the figure that all the hit compounds exhibited two hydrogen bond interactions with the MEK1 receptor, while the reference compound displayed three hydrogen bond interactions with the binding site of MEK1. The iodoalinine moiety of trametinib produces a hydrogen bond interaction with SER 194 of the MEK1 receptor. On the other hand, the cyclopropyl moiety of trametinib makes two hydrogen bond interactions with SER 194 and ASN 195 of the MEK1 receptor. Surprisingly, the quinazoline moiety of the compound DB07642 and methoxy phenyl group DB012661 were producing interactions with LYS 97, which is also an important catalytic residue present in the rooftop of the MEK1 binding pocket. It is also noted that LYS 97 located in the β strand is responsible for the pairing of ATP phosphate to GLU 114 on an adjacent alpha helix [45]. Moreover, the oxygen atom linked with the pyrimidine group of DB12661 makes a hydrogen bond interaction with MET 146, a hinge residue that connects the N and C lobes in the MEK1 receptor [53]. Most importantly, the quinazoline moiety of DB07642 forms an additional hydrogen bond interaction with activation loop residue, such as SER 212, which plays a major role in the phosphorylation of MEK1. It is evident from the literature that most of the MEK 1/2 ligands generate strong interactions with SER 212 [54]. It is important to note that both the lead compounds are bound on the same pattern where the known MEK inhibitors bind. For instance, rafemetinib and RO4987655 interacted with the amino acid residues LYS97 and SER212 of the MEK receptor. On the other hand, CI-1040, PD-0325901, cobimetinib, TAK-733 and GDC-0623 were successfully involved in contact with SER212 of the MEK receptor [6,45]. Based on these pieces of evidence, we are certain that compounds such as DB07642 and DB12661 make strong contact with the functionally important amino acid residues of MEK.

In general, the compound DB012661, also known as urapidil, acts as an antihypertensive drug that inhibits the activity of α-adrenoceptor. It is worth noting that the compound urapidil also resulted in substantial inhibitory activity in several cancer cell lines [55]. On the other hand, the compound DB07642 (5-[1-(2-Fluorobenzyl)piperidin-4-yl]methoxyquinazoline-2,4-diamine) contains crucial pharmacophores. For instance, piperidine, a heterocyclic pharmacophore, has immense importance in the field of drug development. The piperidine derivatives effectively block the several kinase targets (ERK 2, VEGFR 2 and Alb 1) during the in vitro assessment in the liver cancer cell line (HepG2) [56]. Quinazoline is another important pharmacophore that is present in the many approved anticancer drugs, such as erlotinib and vandetanib [57]. Overall, we believe that these compounds may potentially block the activation of MEK, thereby reducing the risk of many malignant effects.

### 3.7. Binding Analysis of Lead Compounds with PIM1

The binding abilities of the lead compounds were also tested on the PIM1 receptor, which is frequently cross-talked with the MAPK pathway. Molecular docking and prime-MM/GBSA analysis of the lead compounds tested against PIM1 were tabulated in Table S5. It is notable that the recently identified dual inhibitor (MEK1 and PIM1) KZ-02 was used as the reference compound in this analysis. The compound KZ-02 obtained a docking score of −4.892 kcal/mol and a binding free energy value of −50.61 kcal/mol. It is notable that both lead compounds displayed better docking scores and binding free energy values than the PIM1 reference compound. The interactions of the lead compounds with the PIM1 receptor were represented in Figure S3. Interestingly, the compound DB07642 displayed three hydrogen bond interactions and 2 pi-pi stacking with the PIM1 receptor. This implies the greater binding potential of the compound DB07642 with the PIM1 receptor. Altogether, we hypothesize that the lead compounds specified in this study may significantly inhibit the activation of both MEK1 and PIM1.

### 3.8. SIE-Based Free Energy of Binding

Since molecular recognition and drug binding have been recognized as dynamic processes, it is thus particularly important to elaborate on the protein–ligand binding capabilities in a presumed dynamic system. To this end, the free energy of binding (Δ*G*_bind_) calculations based on the solvated interaction energy (SIE) were applied and theoretically used to predict the inhibitory activity as it is directly proportional to an experimental inhibitory parameter, K_d_ (${\Delta G}_{\mathrm{bind}}=-\mathrm{RTln}1/Kd$) [58]. Here, the Δ*G*_bind_ values of two focused compounds extracted from the last 10 ns (90–100 ns) snapshots, which were considered to be reaching their equilibrated state (Figure S4), were listed in Table 4 in comparison to the trametinib. The calculated molecular mechanics calculations showed that Van der Waal (vdW) is the main interactive force contributing to the process of molecular complexation of all the focused compounds as well as trametinib (>five to six-fold than electrostatic interaction energy), which corresponds to the molecular docking study by Glide XP. Apart from that, the average Δ*G*_bind_ values of the focused compounds and a reference drug were nearly the same, within the range of −8.4 to −7.5 kcal/mol. In particular, DB12661 possessed a slightly lower Δ*G*_bind_ when compared to the trametinib (Δ*G*_bind_ of −8.41 and −8.17 kcal/mol, respectively), suggesting a minutely higher binding strength than the known drug. On the contrary, compound DB07642 exhibited a slightly higher Δ*G*_bind_ value (Δ*G*_bind_ of −7.52 kcal/mol), which may imply a slight reduction in the ligand binding capability. However, we believed that these two screened compounds could be thermodynamically able to bind to the MEK1 at the ATP-binding site, and both are of particular interest to be subjected to next-step experimental studies, for which DB12661 and DB07642 were rationally considered as a priority and a second top, accordingly.

### 3.9. Key Binding Residues

**Figure 3:**
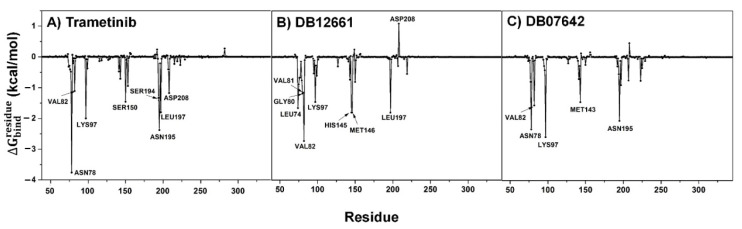
Per-residue decomposition free energy (${\Delta G}_{\mathrm{residue}}^{\mathrm{bind}}$) of the ATPase pocket of MEK1 for the binding of the (**A**,**B**) two screened compounds and (**C**) the known drug, trametinib.

In order to elucidate the key binding amino acid residues within the ATP-binding pocket located at the ATPase domain of MEK1, the decomposition free energy (${\Delta G}_{\mathrm{residue}}^{\mathrm{bind}}$) based upon the MM/GBSA method was computationally predicted, and the total contribution of each amino acid of the known drug and focused complexes was plotted, in which the negative and positive decomposition free energy values manifested the ligand stabilization and destabilization, respectively, as illustrated in Figure 3. It was found that the contributing amino acid residues observed in all the complexes were mainly stabilized through van der Waals (vdW) interactions rather than electrostatic force. This indicates that these two candidate compounds may rely on a mechanism of inhibitory action similar to trametinib. In particular, the amino acids that largely contributed towards the trametinib’s binding (Δ*G* < −1.0 kcal/mol) include ASN78, VAL82, LYS97, SER150, SER194, ASN195, LEU197 and ASP208, of which the SER194 and ASN195 were also found from the docking pose. Among these, ASN78, LYS97 and ASN195 played a pivotal role in the complex stabilization (Δ*G* < −2.0 kcal/mol). In the case of the candidate compounds, it was found that the key amino acid residues contributing to the DB07642 binding are mostly the same residues responsible for trametinib’s binding (ASN78, VAL82, LYS97 and ASN195); one additional residue, M143, was observed. Apart from that, compound DB12661 was primarily stabilized through hydrophobic residue of VAL82 (Δ*G*_bind_ = −2.73 kcal/mol), while seven other residues (LEU74, GLY80, VAL81, LYS97, HIS145, MET146 and LEU197) were also found in the stabilization of the complex via vdW interactions with ${\Delta G}_{\mathrm{residue}}^{\mathrm{bind}}$ in the range of −2.0 to −1.0 kcal/mol. Nevertheless, one negatively charged residue, ASP208, was found to be slightly destabilized; that was probably due to the charge–charge repulsion in the complex system. To sum up, with a higher number of residues largely contributing to DB12661 binding, this compound, as expected, possessed the lowest vdW interactive and total binding free energy (Table 4), where the set of vdW interactions became the main driving force towards the complex formation. On the contrary, some contributing amino acid residues (observed in both trametinib and DB12661) may be somewhat lost during the MEK1–DB07642 complex formation, resulting in the slightly lower Δ*G*_bind_ when compared to the trametinib. We noted that these results are correlated well with the calculated SIE-based Δ*G*_bind_ and each energy component, as listed in Table 4.

### 3.10. Ligand–Protein Hydrogen Bonding

**Figure 4:**
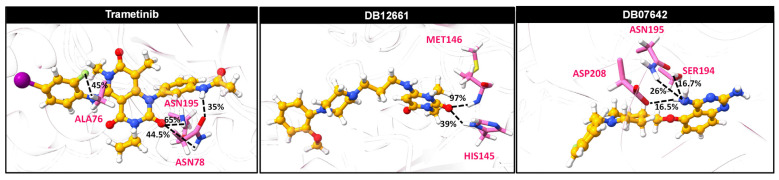
Percentage of hydrogen bond occupations contributing to the binding of two screened compounds (DB12661 and DB07642) and the trametinib within the ATPase domain of MEK1 using two criteria involving the distance and angle between the hydrogen bond donor (HD) and hydrogen acceptor (HA) of ≤3.5 Å for the distance and ≥120° for the angle, respectively.

Hydrogen bonding is one of the non-covalent interactions observed in the formation of protein–ligand complexes and could influence the ligand binding strength. Hence, the intermolecular hydrogen bond interactions were investigated in terms of the percentage of occupations and plotted in Figure 4. As expected, a few strong hydrogen bonds could be observed in the screened compounds and even the trametinib since they are intrinsically hydrophobic ligands. The reference drug trametinib created a strong hydrogen bond with ASN195 (65%), which was also observed by the docking pose (Figure 2). In addition, ALA76 and ASN78 moderately stabilized the drug through 45% and 44.5% of the hydrogen bond occupations, while ASN78 could additionally interact with the drug through 35% of it. For the MEK1–DB12661 complex, we found that the H atom in the backbone (-NH_2_) of MET146 exhibited a very strong hydrogen bond, while the polar H atom in the imidazole ring of HIS145 showed a moderate level. In the case of DB07642, there are three amino acid residues stabilizing the DB07642 binding, which include ASN195, ASP208 and SER194. Among these, the H atom in the amino side chain of ASN195 displayed the highest chance of hydrogen bond occurrence with percentage occupations of 26%, while the other two residues merely exhibited a weak hydrogen bond (≈17%). Altogether, these obtained results suggested that the intermolecular hydrogen bond interactions did not play a major role responsible for the complex stabilization observed in all the studied compounds, including the trametinib. On the other hand, the ligand binding within the ATP-binding pocket of MEK1 was predominantly contributed by vdW interactions, as discussed previously.

## 4. Conclusions

In conclusion, the DrugBank compounds were screened through the different computational approaches to discover the potential MEK inhibitors. Initially, molecular docking and various scoring functions were implemented to screen the active molecules against the MEK protein. Overall, the screening demonstrated that compounds such as DB07642 and DB12661 were able to tightly bind with the MEK receptor. Notably, the presence of crucial pharmacophore moieties in the hit compounds gives additional support to their inhibitory activity. In addition, the modes of action of these compounds were comprehended through the connection of the ligand with the MEK active segment residues. Most importantly, the compounds’ inhibitory activity was also examined with the PIM1 receptor since it upregulated during the action of several MEK inhibitors. Further, the MD simulation and end-point free energy calculation validated the binding mode of the lead compounds with the MEK receptor. Thus, we hypothesize that further experimental validation of our research findings will help to level up the cancer treatment in the near future.

## Figures and Tables

**Table 1:** The top five binding sites of MEK1 receptor predicted by sitemap.

Sites	Site Score	Dscore	Binding Pocket Region
1	1.067	0.995	LEU74, GLY75, ALA76, GLY77, ASN78, GLY79, GLY80, VAL82, ALA95, LYS97, ILE99, VAL127, MET143, GLU144, HIS145, MET146, GLY149, SER150, ASP152, GLN153, LYS192, SER194, ASN195, LEU197, CYS207, ASP208, PHE209, GLY210, VAL211, SER212
2	1.028	1.05	GLU39, GLN45, GLN46, ARG49, LEU50, ALA52, PHE53, LEU54, GLN56, LYS57, LEU92, VAL93, HIS119, GLU120, CYS121, ASN122, SER123, PRO124, TYR125, ILE126, VAL127, GLY128, PHE129, TYR130, GLU144, HIS145, MET146, ASP147, LYS168, ILE171, ALA172, LYS175, ASN199, ARG201, GLY202, GLU203, ILE204, LYS205, ASP365, VAL369, ASP370, PHE371, ALA372
3	0.974	1.005	GLU39, LEU40, GLU41, LEU42, GLN46, ASN122, SER123, PRO124, TYR125, ILE174, LYS175, THR178, TYR179, ARG181, GLU182, LYS183, VAL242, LEU352, LYS353, MET356
4	0.819	0.782	LEU118, HIS119, ILE126, LEU180, HIS184, LYS185, ILE186, MET187, HIS188, ARG189, ASP208, PHE209, GLY210, GLY213, GLN214, ASP217
5	0.702	0.673	VAL254, VAL258, PRO262, PRO265, PRO266, LEU271, PRO321, PRO322, PRO323, LYS324, LEU325, PRO326, SER327, GLN335, ASN339

**Table 2:** Molecular docking and binding free energy calculations of hit compounds against MEK1 receptor.

Compound ID	Docking Score (kcal/mol)	Δ*G*_bind_ (kcal/mol)	Δ*G*_bind_ Coulomb	Δ*G*_bind_ Lipophilic	Δ*G*_bind_ Solv GB	Δ*G*_bind_ vdW	Ligand Strain Energy
Reference	−3.423	−46.137	−13.639	−32.888	32.888	−43.528	24.43
DB12661	−7.051	−87.013	−16.84	−46.647	31.692	−57.476	5.29
DB07642	−6.174	−83.845	−20.352	−42.431	28.151	−55.062	8.453
DB02366	−7.427	−76.925	−34.282	−36.488	39.865	−47.657	7.09
DB08251	−11.98	−75.956	−34.186	−24.74	27.909	−44.926	3.995
DB01771	−7.775	−75.093	−28.532	−45.543	38.739	−46.271	10.615
DB12847	−6.716	−66.948	−29.293	−28.799	31.254	−41.632	4.669
DB07177	−6.989	−65.876	−14.264	−51.153	31.763	−39.082	18.693
DB13174	−9.287	−64.939	−22.947	−21.409	20.618	−42.359	2.315
DB07125	−8.416	−63.963	−20.194	−26.628	25.206	−42.305	8.554
DB07773	−9.256	−61.255	−31.925	−29.541	32.325	−36.44	7.628
DB07546	−6.456	−61.064	−24.4	−37.67	35.031	−36.04	9.162
DB02849	−8.72	−59.793	−49.808	−16.914	42.084	−35.493	5.028
DB02709	−7.091	−59.576	−21.878	−29.309	21.114	−32.041	3.817
DB04241	−8.469	−57.965	−46.177	−23.207	30.706	−27.2	10.366

**Table 3:** 2D structure of hit compounds with their predicted ADME properties.

DrugBank ID	2D Strucure	Stars ^a^	CNS ^b^	QPlogS ^c^	HOA ^d^
Reference	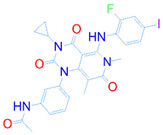	1	−2	−8.042	1
DB08251	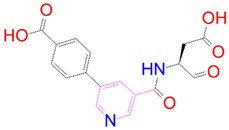	1	−2	−3.274	1
DB13174	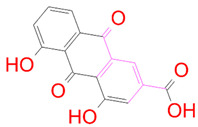	0	−2	−2.449	2
DB07773	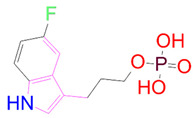	0	−2	−1.457	1
DB02849	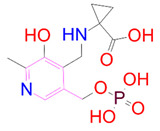	1	−2	−2.647	2
DB04241	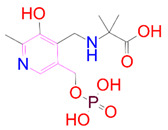	0	−2	−3.902	2
DB07125	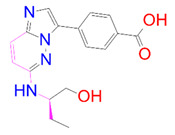	0	−2	−1.666	1
DB01771	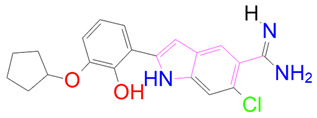	0	−2	−2.794	3
DB02366	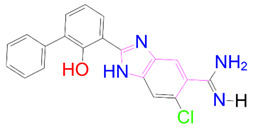	0	−2	−5.171	3
DB02709	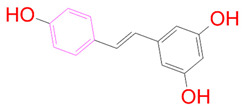	0	−2	−0.905	2
DB12661	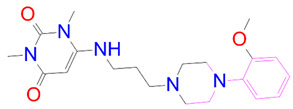	0	0	−5.177	3
DB07177	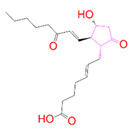	0	−2	−4.861	3
DB12847	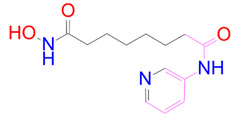	0	−2	−3.52	2
DB07546	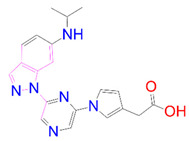	0	−2	−5.71	3
DB07642	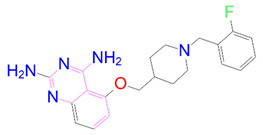	0	−1	−3.874	3

^a^—The number of attributes or descriptor values that are beyond the 95% range of similar values for identified drugs; ^b^—predicted central nervous system activity; ^c^—predicted aqueous solubility; ^d^—Human Oral Absorption; pink color indications in the 2D structure represent the 2D structure alignment of the compounds against the reference compound.

**Table 4:** Average Δ*G*_bind_ values (kcal/mol) of focused compounds as well as trametinib in complex with MEK1 calculated by the SIE method using α = 0.105, γ = 0.013 and C = −2.89, respectively.

Compounds	Energy Components				
	*E*_vdW_	*E*_ele_	Reaction Field	Cavity	Δ*G*_bind_
Trametinib	−51.05 ± 0.34	−9.58 ± 0.20	19.25 ± 0.26	−9.05 ± 0.07	−8.17 ± 0.04
DB12661	−52.08 ± 0.32	−4.29 ± 0.17	12.18 ± 0.24	−8.52 ± 0.05	−8.41 ± 0.04
DB07642	−43.91 ± 0.37	−6.90 ± 0.21	14.62 ± 0.36	−8.02 ± 0.06	−7.52 ± 0.04

## Data Availability

Not applicable.

## Supplementary Materials

The following supporting information can be downloaded at: https://www.mdpi.com/article/10.3390/pharmaceutics14010059/s1. Table S1: Predicted pKa values of each amino acid residues at different conditions. Table S2: Compounds used for validation of docking and RF-Score-VS. Table S3: Multiple screening analysis of the compounds against MEK1. Table S4: Three-fold validation on glide docking analysis of hit compounds. Table S5: Molecular docking and binding free energy calculations of top hit compounds against PIM1 receptor. Figure S1: ROC analysis of screening methods. (a) Docking; (b) RF-Score-VS. Figure S2: Binding frequency of the ligand molecules on top three predicted binding sites. The coloured dots represent the binding sites: site 1 (red), site 2 (orange), and site 3 (yellow). The coloured chemical structures depict ligand molecules binding positions on various binding sites. Ligand bound in site 1 (purple); site 2 (green); site 3 (sky blue). Figure S3: Ligand interaction diagram of hit compounds (a) KZ-02 (Reference); (b) DB012661; (c) DB07642 with PIM1 receptor. Figure S4: Root-mean-square displacement (RMSD) plot for the backbone amino acid residues within a 5-Å sphere around the ligand. The data were derived from the three independent runs with different initial velocities.
