# Supplementary material for: In Silico Screening of Available Drugs Targeting Non-Small Cell Lung Cancer Targets: A Drug Repurposing Approach
Source: Pharmaceutics. 2021 Dec 28;14(1):59. doi: 10.3390/pharmaceutics14010059 (PMC8778223; doi:10.3390/pharmaceutics14010059)
Supplement: Supplementary file 1 [file pharmaceutics-14-00059-s001.zip › pharmaceutics-1423271-supplementary.pdf]

pharmaceutics

Supplementary Materials: In Silico Screening of Available Drugs Targeting Non-Small Cell Lung Cancer Targets: A Drug Repurposing Approach

Table S1. Predicted pKa values of each amino acid residues at different conditions.

Amino Acid Residues	pKa (Before Minimization)	pKa (After Minimization)	Corrected Values
GLU39	7.94	7.92	0.02
GLU39	4.49	4.55	−0.06
GLU41	4.55	4.56	−0.01
GLU44	4.88	4.89	−0.01
ARG47	12.67	12.51	0.16
LYS48	10.55	10.56	−0.01
ARG49	12.9	12.83	0.07
GLU51	4.12	4.27	−0.15
LYS57	10.06	9.84	0.22
LYS59	10.31	10.35	−0.04
GLU62	4.56	4.55	0.01
LYS64	11.94	11.72	0.22
ASP65	3.16	3.18	−0.02
ASP66	3.88	4.26	−0.38
ASP67	2.88	2.82	0.06
GLU69	4.14	3.71	0.43
LYS70	10.3	10.28	0.02
GLU73	4.74	4.74	0
LYS84	10.24	10.2	0.04
HIE87	5.48	5.47	0.01
LYS88	10.72	10.72	0
ARG96	12.41	12.48	−0.07
LYS97	9.78	9.66	0.12
HIE100	6.53	6.43	0.1
GLU102	4.09	4.04	0.05
LYS104	10.25	10.27	−0.02
ARG108	12.82	12.69	0.13
ARG113	12.31	12.3	0.01
GLU114	5.02	5.08	−0.06
HIS119	6.89	6.98	−0.09
GLU120	3.78	4.15	−0.37
CYS121	11.61	11.62	−0.01
TYR125	9.93	9.66	0.27
TYR130	12.17	12.18	−0.01
TYR134	10.25	10.45	−0.2
ASP136	4.04	4.06	−0.02
GLU138	4.8	4.93	−0.13
CYS142	12	12.02	−0.02
GLU144	4.56	4.65	−0.09
HIE145	4.95	4.9	0.05
ASP147	3.09	3.29	−0.2
ASP152	3.7	3.66	0.04
LYS156	10.54	10.55	−0.01
LYS157	10.36	10.35	0.01
ARG160	11.9	11.86	0.04
GLU163	3.64	3.7	−0.06
LYS168	9.67	9.62	0.05

LYS175	10.96	11.02	−0.06
TYR179	11.15	11.41	−0.26
ARG181	12.32	12.13	0.19
GLU182	4.79	4.82	−0.03
LYS183	10.48	10.45	0.03
HIE184	5.65	5.27	0.38
LYS185	7.95	7.76	0.19
HIE188	6.29	6.13	0.16
ARG189	10.82	10.54	0.28
ASP190	4.7	4.74	−0.04
LYS192	9.86	9.87	−0.01
ARG201	12.16	12.14	0.02
GLU203	2.84	3.06	−0.22
LYS205	10.94	10.8	0.14
CYS207	11.44	11.68	−0.24
ASP208	2.61	2.26	0.35
ASP217	4.3	4.29	0.01
ARG227	13.12	13.02	0.1
TYR229	9.46	9.56	−0.1
GLU233	4.28	3.72	0.56
ARG234	13.08	12.91	0.17
HIE239	6.4	6.38	0.02
TYR240	9.88	9.99	−0.11
ASP245	5.69	6.46	−0.77
GLU255	6.05	6.1	−0.05
ARG260	12.1	11.97	0.13
TYR261	11.3	11.3	0
ASP267	3.52	3.45	0.07
LYS269	10.53	10.52	0.01
GLU270	4.71	4.73	−0.02
GLU272	4.73	4.74	−0.01
CYS277	9.17	9.19	−0.02
ARG305	12	12	0
GLU312	3.99	3.99	0
ASP315	4.1	4.16	−0.06
TYR316	10.76	10.83	−0.07
GLU320	4.65	4.68	−0.03
LYS324	10.2	10.15	0.05
GLU333	3.26	3.42	−0.16
ASP336	2.41	2.73	−0.32
LYS340	11.21	10.82	0.39
CYS341	12.6	12.51	0.09
LYS344	10.02	9.99	0.03
GLU348	4.66	4.64	0.02
ARG349	11.13	11.14	−0.01
ASP351	2.37	2.92	−0.55
LYS353	11.54	11.16	0.38
HIE358	6.03	6.04	−0.01
LYS362	10.34	10.3	0.04
ARG363	14.77	14.17	0.6
ASP365	3.73	3.89	−0.16
GLU367	4.07	4.44	−0.37
GLU368	4.53	4.53	0
ASP370	3.37	3.41	−0.04
CYS376	9.74	9.5	0.24

Table S2. Compounds used for validation of docking and RF-Score-VS.

Compounds Name	Docking Score (kcal/mol)	RF−Score−VS	Activity (nm)	Type
CHEMBL33906	−9.821	5.958	47	Active
CHEMBL30790	−9.672	6.004	14	Active

CHEMBL281662	−9.396	6.125	18	Active
CHEMBL31143	−9.353	6.003	20	Active
CHEMBL287959	−9.23	5.994	76	Active
CHEMBL214683	−9.165	6.062	43	Active
CHEMBL34072	−9.085	5.953	25	Active
CHEMBL290886	−8.916	5.964	55	Active
CHEMBL216832	−8.823	6.085	44	Active
CHEMBL31497	−8.655	6.19	26	Active
CHEMBL217130	−8.532	5.97	100	Active
CHEMBL282361	−8.519	5.975	21	Active
CHEMBL34436	−8.444	5.983	170	Active
CHEMBL320688	−8.442	6.102	212	Active
CHEMBL535	−7.798	5.972	130	Active
CHEMBL31639	−7.508	6	160	Active
CHEMBL201833	−7.22	6.072	386	Active
CHEMBL436137	−7.118	6.074	69	Active
CHEMBL37548	−6.838	5.956	540	Active
CHEMBL422940	−6.612	5.97	410	Active
CHEMBL329492	−6.587	6.06	300	Active
CHEMBL380328	−5.746	5.996	410	Active
CHEMBL201384	−5.551	6.105	361	Active
CHEMBL382616	−5.544	6.139	168	Active
CHEMBL322395	−4.99	6.136	280	Active
ZINC00104965	−3.665	5.979	−	Decoy
ZINC00302473	−5.494	5.96	−	Decoy
ZINC00510305	−6.082	5.973	−	Decoy
ZINC01311479	−4.827	5.963	−	Decoy
ZINC01808151	−6.417	5.979	−	Decoy
ZINC02356139	−5.859	5.972	−	Decoy
ZINC02474446	−5.235	5.96	−	Decoy
ZINC03173966	−2.574	5.956	−	Decoy
ZINC04268246	−5.063	5.962	−	Decoy
ZINC04685857	−6.638	5.961	−	Decoy
ZINC05275925	−6.043	5.966	−	Decoy
ZINC05276752	−2.923	5.964	−	Decoy
ZINC05463577	−3.581	5.968	−	Decoy
ZINC05833428	−4.762	5.96	−	Decoy
ZINC05833428	−3.559	5.958	−	Decoy
ZINC05941968	−6.446	5.974	−	Decoy
ZINC06418307	−5.699	5.967	−	Decoy
ZINC06419602	−2.972	5.958	−	Decoy
ZINC06529050	−6.323	5.972	−	Decoy
ZINC06584512	−6.402	5.968	−	Decoy
ZINC06685196	−5.916	5.971	−	Decoy
ZINC09446432	−6.158	5.959	−	Decoy
ZINC11294859	−5.919	5.968	−	Decoy
ZINC13130028	−4.542	5.963	−	Decoy
ZINC13130028	−5.457	5.958	−	Decoy
ZINC13262863	−4.169	5.956	−	Decoy
ZINC13523686	−3.452	5.957	−	Decoy
ZINC14271552	−6.661	5.977	−	Decoy
ZINC14460259	−4.748	5.971	−	Decoy
ZINC14547785	−6.253	5.96	−	Decoy
ZINC14806183	−4.482	5.973	−	Decoy
ZINC17002670	−2.431	5.965	−	Decoy
ZINC17054333	−6.416	5.961	−	Decoy
ZINC18196734	−3.918	5.96	−	Decoy
ZINC19767181	−6.57	5.975	−	Decoy
ZINC20343524	−6.42	5.964	−	Decoy
ZINC21238631	−5.073	5.967	−	Decoy
ZINC26895232	−6.396	5.963	−	Decoy
ZINC30884190	−5.167	5.963	−	Decoy

ZINC35276985	−6.088	5.972	−	Decoy
ZINC35698004	−5.537	5.958	−	Decoy
ZINC37452574	−5.498	5.958	−	Decoy
ZINC37552340	−3.395	5.962	−	Decoy
ZINC39280364	−5.187	5.972	−	Decoy
ZINC39465282	−6.358	5.959	−	Decoy
ZINC40282812	−5.544	5.959	−	Decoy
ZINC40338438	−6.341	5.961	−	Decoy
ZINC40728537	−2.493	5.967	−	Decoy
ZINC42175424	−5.278	5.957	−	Decoy
ZINC42652324	−6.421	5.957	−	Decoy
ZINC43554560	−5.503	5.969	−	Decoy
ZINC43666562	−6.276	5.968	−	Decoy
ZINC43666583	−4.237	5.957	−	Decoy
ZINC43668535	−6.076	5.958	−	Decoy
ZINC44069419	−5.471	5.96	−	Decoy
ZINC44888470	−6.526	5.963	−	Decoy
ZINC45112290	−6.361	5.958	−	Decoy
ZINC45728136	−7.393	5.979	−	Decoy
ZINC49067143	−3.803	5.963	−	Decoy
ZINC50306995	−6.372	5.973	−	Decoy
ZINC51036903	−5.935	5.959	−	Decoy
ZINC51088673	−6.433	5.961	−	Decoy
ZINC52902235	−5.997	5.96	−	Decoy
ZINC55179637	−6.667	5.963	−	Decoy
ZINC57316590	−8.039	5.96	−	Decoy
ZINC58279609	−4.188	5.97	−	Decoy
ZINC58279616	−6.008	5.964	−	Decoy
ZINC58279785	−4.669	5.965	−	Decoy
ZINC61534036	−6.278	5.962	−	Decoy
ZINC61589830	−6.541	5.974	−	Decoy
ZINC61819155	−6.334	5.961	−	Decoy
ZINC62766937	−5.291	5.961	−	Decoy
ZINC62843866	−6.155	5.958	−	Decoy
ZINC62870625	−4.95	5.966	−	Decoy
ZINC66739067	−4.906	5.967	−	Decoy

Table S3. Multiple screening analysis of the compounds against MEK1.

Compound ID	Docking Score (kcal/mol)			RF−Score−VS	Tanimoto Coefficient
	Site 1	Site 2	Site 3		
Reference	−3.423	−1.706	−3.65	6.656	
DB00147	−6.382	−6.709	−4.149	7.098	0.614278103
DB00168	−6.582	−3.431	−4.283	7.103	0.676993398
DB00190	−9.839	−6.646	−6.306	7.105	0.684580613
DB00816	−7.836	−4.489	−3.763	7.1	0.636269964
DB00871	−8.309	−4.796	−3.834	7.1	0.636269964
DB01088	−7.395	−4.438	−5.369	7.101	0.667546174
DB01157	−6.278	−4.591	−5.726	7.098	0.616965227
DB01291	−7	−6.408	−4.595	7.099	0.634309194
DB01634	−6.625	−5.758	−4.947	7.098	0.606076759
DB01685	−6.757	−4.354	−4.112	7.098	0.616965227
DB01758	−8.801	−6.65	−5.717	7.098	0.634121274
DB01771	−7.775	−4.624	−4.958	7.098	0.634121274
DB02041	−7.438	−4.049	−4.927	7.098	0.634121274
DB02088	−6.953	−3.715	−4.92	7.117	0.707329317
DB02142	−7.657	−6.217	−5.27	7.098	0.603510583
DB02209	−8.651	−9.064	−5.669	7.099	0.635107472
DB02215	−7.442	−4.439	−3.648	7.098	0.601807549
DB02256	−7.709	−5.649	−3.976	7.1	0.655995935
DB02268	−7.385	−4.7	−5.493	7.105	0.687088608
DB02366	−7.427	−3.644	−5.082	7.098	0.631876607

DB02408	−6.205	−3.61	−6.334	7.098	0.600620797
DB02475	−6.914	−5.321	−3.746	7.098	0.631551901
DB02652	−7.502	−4.803	−5.248	7.103	0.681725888
DB02709	−7.091	−5.624	−4.745	7.128	0.709984947
DB02725	−6.527	−5.823	−4.117	7.098	0.631551901
DB02842	−6.339	−5.758	−4.658	7.098	0.631551901
DB02849	−8.72	−7.285	−5.801	7.1	0.667002519
DB02989	−7.364	−3.901	−4.831	7.098	0.63062597
DB03037	−9.307	−5.124	−5.808	7.108	0.691612255
DB03285	−8.281	−5.81	−5.772	7.105	0.684580613
DB04058	−7.813	−4.262	−5.227	7.103	0.681564246
DB04169	−7.527	−4.138	−5.075	7.098	0.625527426
DB04241	−8.469	−5.572	−6.386	7.1	0.655789474
DB04267	−7.67	−4.665	−4.78	7.1	0.655789474
DB04324	−6.833	−4.768	−4.102	7.1	0.639504899
DB04598	−7.143	−5.346	−5.574	7.126	0.707329317
DB06255	−10.304	−6.508	−6.288	7.1	0.65394402
DB06397	−6.676	−3.995	−4.589	7.098	0.615995763
DB06529	−7.131	−3.654	−3.81	7.098	0.615995763
DB06762	−6.674	−3.904	−4.056	7.098	0.610115911
DB06819	−6.25	−4.127	−4.447	7.103	0.677664975
DB06832	−7.715	−4.441	−4.752	7.099	0.635107472
DB06873	−6.334	−3.907	−4.815	7.1	0.660030628
DB06997	−6.582	−3.818	−5.672	7.098	0.623777663
DB07125	−8.416	−4.498	−5.051	7.098	0.623667377
DB07177	−6.989	−5.47	−4.796	7.103	0.680786687
DB07546	−6.456	−5.852	−4.423	7.098	0.623188406
**DB07642**	**−6.174**	**−7.065**	**−6.817**	**7.098**	**0.622313204**
DB07718	−7.426	−5.062	−5.071	7.105	0.684580613
DB07773	−9.256	−5.543	−5.521	7.1	0.654545455
DB07795	−9.253	−5.545	−5.643	7.1	0.65394402
DB07854	−8.951	−4.224	−4.168	7.098	0.620600414
DB07855	−8.871	−3.969	−4.218	7.098	0.620600414
DB08191	−8.376	−4.259	−5.016	7.098	0.620298815
DB08251	−11.98	−5.367	−4.919	7.1	0.655789474
DB08350	−7.279	−5.485	−3.922	7.098	0.620298815
DB08523	−6.131	−4.084	−4.329	7.104	0.684343434
DB08660	−9.369	−7.006	−6.804	7.105	0.684580613
DB08846	−10.807	−5.335	−6.228	7.1	0.65394402
DB11673	−6.215	−5.389	−4.369	7.098	0.615995763
DB11830	−6.173	−5.165	−4.774	7.108	0.695585997
**DB12661**	**−7.051**	**−7.569**	**−6.982**	**7.098**	**0.615995763**
DB12818	−8.106	−6.325	−4.401	7.1	0.651553744
DB12847	−6.716	−6.128	−4.515	7.098	0.615942029
DB13174	−9.287	−7.87	−5.633	7.1	0.65394402
DB13421	−8.6	−6.357	−4.412	7.103	0.681725888

Highlighted compound indicates the lead compound considered in this analysis.

Table S4. Three-fold validation on glide docking analysis of hit compounds.

Compounds	1st Iteration	2nd Iteration	3rd Iteration	Average Docking Score (kcal/mol)
Trametinib	−3.423	−4.153	−2.76	−3.445
DB08251	−11.98	−12.371	−12.358	−12.23
DB13174	−9.287	−9.62	−10.667	−9.858
DB07773	−9.256	−9.205	−9.207	−9.222
DB02849	−8.72	−9.038	−9.038	−8.932
DB04241	−8.469	−8.614	−8.16	−8.414
DB07125	−8.416	−8.207	−8.117	−8.246
DB01771	−7.775	−7.346	−7.346	−7.489
DB02366	−7.427	−7.241	−7.139	−7.269
DB02709	−7.091	−7.134	−7.062	−7.095

DB12661	−7.051	−7.062	−7.029	−7.043
DB07177	−6.989	−6.754	−6.982	−6.90
DB12847	−6.716	−6.152	−6.152	−6.34
DB07546	−6.456	−5.874	−5.874	−6.068
DB07642	−6.174	−5.827	−5.827	−5.942

Table S5. Molecular docking and binding free energy calculations of top hit compounds against PIM1 receptor.

Compounds Name	Docking Score (kcal/mol)	ΔG_{Bind} (kcal/mol)	ΔG_{Bind} Coulomb	ΔG_{Bind} Hbond	ΔG_{Bind} Solv GB	ΔG_{Bind} vdW	Lig Strain Energy
KZ−02	−4.892	−50.61	30.58	−1.22	12.05	−44.55	6.55
DB012611	−5.594	−68.29	−18.72	−3.12	36.37	−42.29	13.013
DB07642	−5.502	−55.97	−12.58	−2.73	32.97	−38.67	7.522

(a)

(b)

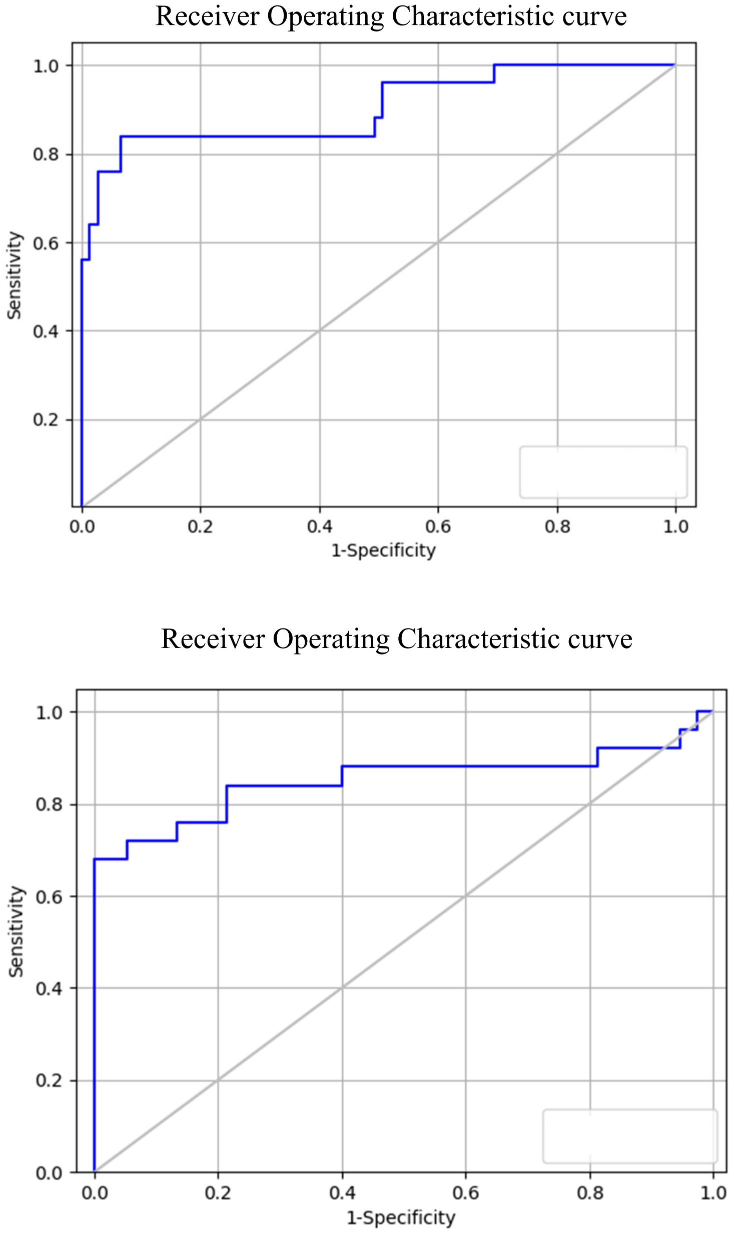

Figure S1. ROC analysis of screening methods. (**a**) Docking; (**b**) RF-Score-VS.

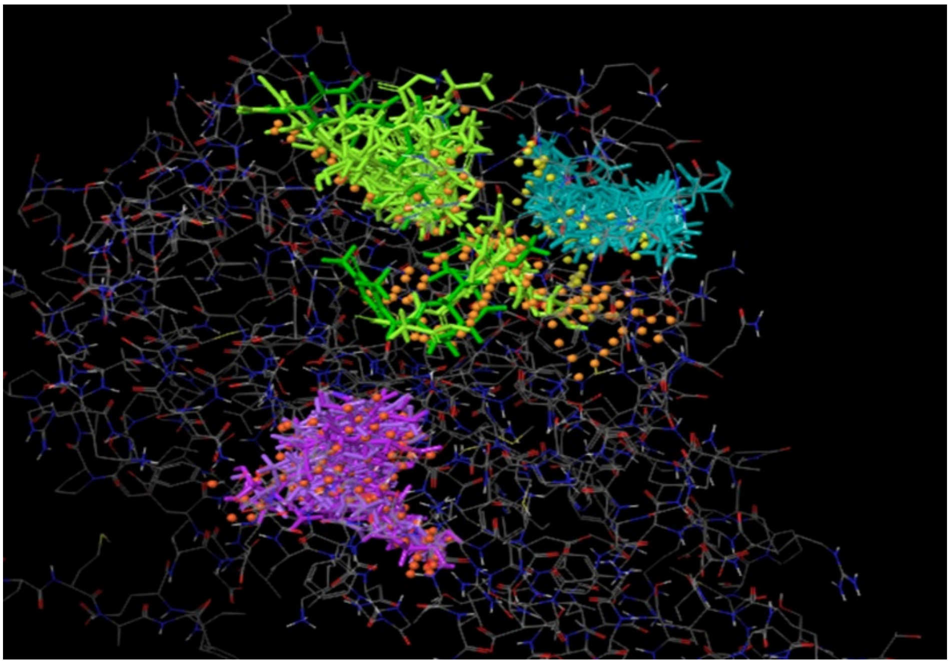

Figure S2. Binding frequency of the ligand molecules on top three predicted binding sites. The coloured dots represent the binding sites: site 1 (red), site 2 (orange), and site 3 (yellow). The coloured chemical structures depict molecules binding positions on various binding sites. Ligand bound in site 1 (purple); site 2 (green); site 3 (sky blue).

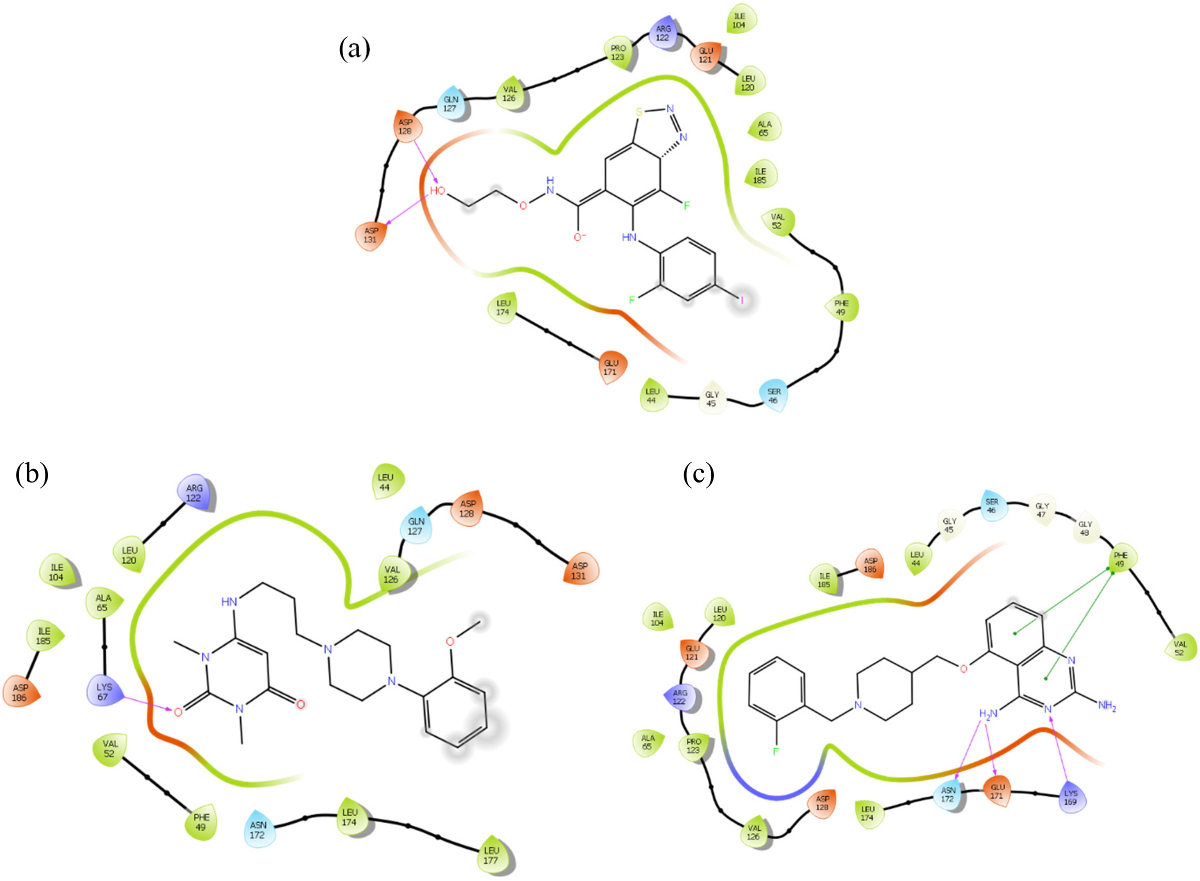

Figure S3. Ligand interaction diagram of hit compounds (**a**) KZ-02 (Reference); (**b**) DB012661; (**c**) DB07642 with PIM1 receptor.

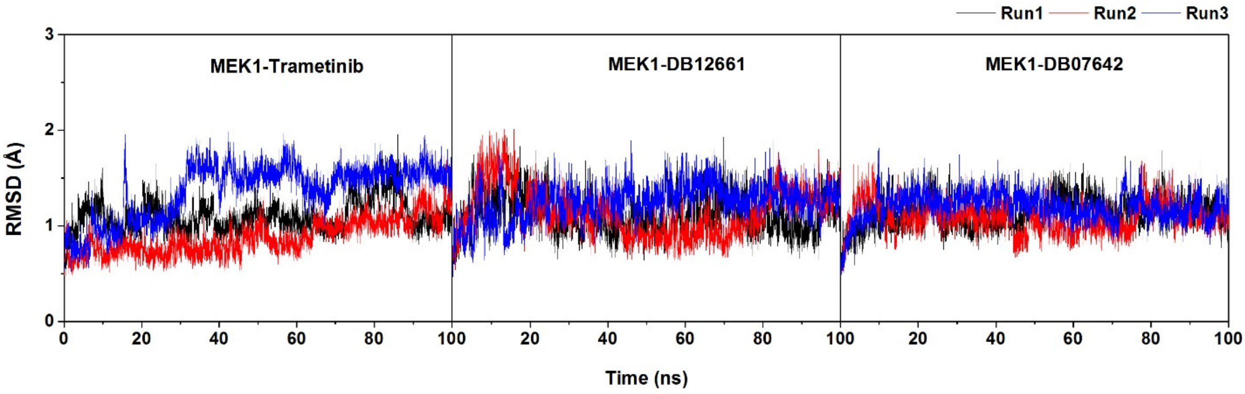

Figure S4. Root-mean-square displacement (RMSD) plot for the backbone amino acid residues within a 5-Å sphere around the ligand. The data were derived from the three independent runs with different initial velocities.
